# Problems in the management of Tokyo 2020 Paralympic Games cycling events at Fuji International Speedway during the COVID‐19 pandemic

**DOI:** 10.1002/ams2.754

**Published:** 2022-05-17

**Authors:** Youichi Yanagawa

**Affiliations:** ^1^ Department of Acute Critical Care Medicine, Shizuoka Hospital Juntendo University Izunokuni City Japan

Dear Editor,

I write to report my department’s experience of the Tokyo 2020 Paralympic Games cycling events.^1^ The Tokyo 2020 Paralympic Games cycling events at Fuji International Speedway (FIS) at Gotemba City in eastern Shizuoka Prefecture, Japan, were held without spectators from August 31, 2021 to September 3, 2021. Time trial races were held on the first day of the cycling events, while road races were held on the second to fourth days. Fuji International Speedway is licensed as a grade 1 circuit by the Federation Internationale de l'Automobile. Hospital A at Gotemba City was engaged as a designated hospital by the Tokyo 2020 Organizing Committee (TOC) for the Olympic and Paralympic Games. However, Hospital A did not have an acute critical care center and could not treat patients with multiple severe injuries. In addition, Hospital A could not receive emergency patients due to an outbreak of COVID‐19 that occurred in the hospital just before the Paralympic Games cycling events; thus, Hospital A lost its function as the designated hospital for the Paralympic Games cycling events at FIS. In that time, Shizuoka Prefecture experienced its fifth and largest wave of the COVID‐19 pandemic, which had started after the Tokyo 2020 Olympic Games, even though Tokyo was under a state of emergency (Fig. 1). During peacetime, patients involved in car accidents at the FIS are managed by a physician‐staffed helicopter (helicopter emergency medical service [HEMS]) operated by Juntendo Shizuoka Hospital (JSH), which has a level 1 acute critical care center because such patients have a high risk of having multiple severe injuries.^2^, ^3^ The hospital is located in eastern Shizuoka Prefecture and is the leader of the medical control council system, which oversees the activities of the Gotemba Fire Department, as a member of the Japanese Medical Association.^2^, ^3^, ^4^ No official plan was established by the TOC in preparation for the unlikely situation where the designated hospital could not fulfill its function, thus requiring JSH to provide backup assistance in the event that Hospital A was unable to handle any patients with severe injuries. However, JSH regularly provides such backup assistance. During the Tokyo 2020 Paralympic Games cycling events at FIS, three Paralympians with fall‐related severe injuries were directly transported to JSH by ground ambulance, which took approximately 60 min. Gotemba Fire Department requested the dispatch of the Eastern Shizuoka HEMS, however, the HEMS was not available due to other missions. The patients (male, *n* = 2; female, *n* = 1) were aged in their 40s to 60s. The diagnoses were cerebral concussion (*n* = 1), traumatic subarachnoid hemorrhage with elbow avulsion (*n* = 1), and pneumothorax with lung contusion (*n* = 1). All three Paralympians required admission to JSH, and fortunately obtained favorable outcomes and safely returned to their own nation. Fall‐related accidents that extraordinary athletes experience during cycling events could result in fatal or multiple severe injuries.^1^ Accordingly, hospitals that are designated for the management of such athletes should be able to treat such patients to obtain a favorable outcome.^5^ In addition, a backup designated hospital is necessary in the event of infectious disease outbreaks, such as the COVID‐19 pandemic. Unfortunately, no official post‐event verification was carried out by the TOC after the Olympic and Paralympic Games.

**Fig 1:**
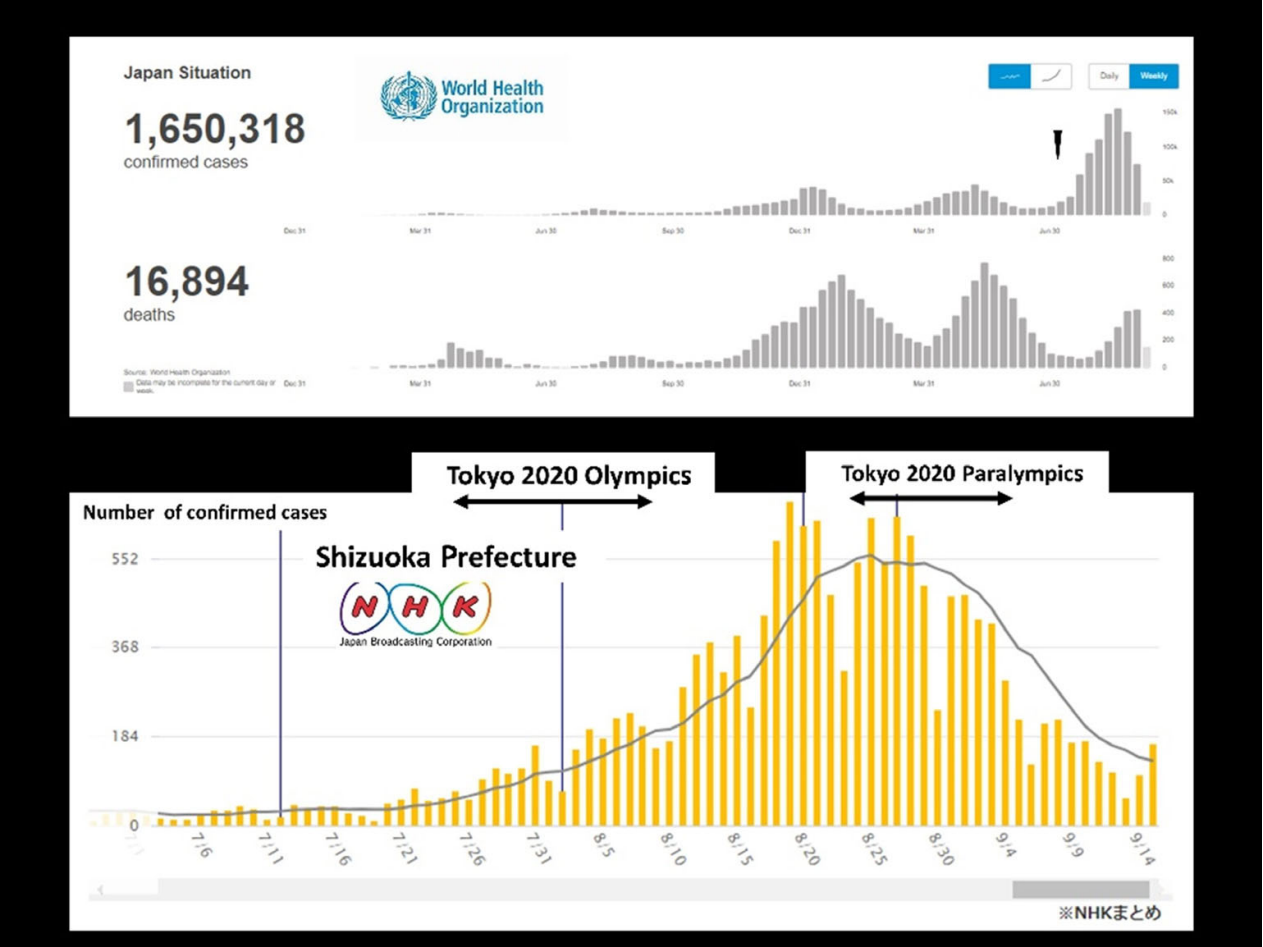
Trends in the number of patients in Japan with COVID‐19. The top figure shows the trends in Japan from January 2020 to September 2021, as presented by the World Health Organization. Japan experienced its fifth and largest wave after the commencement of the Tokyo 2020 Olympic Games (black triangle). The lower figure shows trends in Shizuoka Prefecture, Japan, from July 2021 to September 2021, as presented by Japan Broadcasting Corporation. Shizuoka also experienced its fifth and largest wave after the commencement of the Tokyo 2020 Olympic Games.

## DISCLOSURE

Conflict of interest: None.
